# Locally advanced rectal cancer: 3D diffusion-prepared stimulated-echo turbo spin-echo *versus* 2D diffusion-weighted echo-planar imaging

**DOI:** 10.1186/s41747-019-0138-x

**Published:** 2020-02-07

**Authors:** Qinwei Zhang, Petra J. van Houdt, Doenja M. J. Lambregts, Baukelien van Triest, Marnix P. M. Kop, Bram F. Coolen, Gustav J. Strijkers, Uulke A. van der Heide, Aart J. Nederveen

**Affiliations:** 1 Amsterdam UMC, Radiology and Nuclear Medicine, University of Amsterdam, Room Z0-178, Meibergdreef 9, 1100 DD Amsterdam, Netherlands; 2 Department of Radiation Oncology, Netherlands Cancer Institute, Amsterdam, Netherlands; 3 Department of Radiology, Netherlands Cancer Institute, Amsterdam, Netherlands; 4 Amsterdam UMC, Biomedical Engineering and Physics, University of Amsterdam, Amsterdam, the Netherlands

**Keywords:** Diffusion magnetic resonance imaging, Echo-planar imaging, Magnetic resonance imaging, Neoplasm staging, Rectal neoplasms

## Abstract

**Background:**

Diffusion-weighted imaging (DWI) has shown great value in rectal cancer imaging. However, traditional DWI with echo-planar imaging (DW-EPI) often suffers from geometrical distortions. We applied a three-dimensional diffusion-prepared stimulated-echo turbo spin-echo sequence (DPsti-TSE), allowing geometrically undistorted rectal DWI. We compared DPsti-TSE with DW-EPI for locally advanced rectal cancer DWI.

**Methods:**

For 33 prior-to-treatment patients, DWI images of the rectum were acquired with DPsti-TSE and DW-EPI at 3 T using b-values of 200 and 1000 s/mm^2^. Two radiologists conducted a blinded scoring of the images considering nine aspects of image quality and anatomical quality. Tumour apparent diffusion coefficient (ADC) and distortions were compared quantitatively.

**Results:**

DPsti-TSE scored significantly better than DW-EPI in rectum distortion (*p =* 0.005) and signal pileup (*p =* 0.001). DPsti-TSE had better tumour Dice similarity coefficient compared to DW-EPI (0.84 *versus* 0.80, *p =* 0.010). Tumour ADC values were higher for DPsti-TSE compared to DW-EPI (1.47 *versus* 0.86 × 10^-3^ mm^2^/s, *p <* 0.001). Radiologists scored DPsti-TSE significantly lower than DW-EPI on aspects of overall image quality (*p =* 0.001), sharpness (*p <* 0.001), quality of fat suppression (*p <* 0.001), tumour visibility (*p =* 0.009), tumour conspicuity (*p =* 0.010) and rectum wall visibility (*p =* 0.005).

**Conclusions:**

DPsti-TSE provided geometrically less distorted rectal cancer diffusion-weighted images. However, the image quality of DW-EPI over DPsti-TSE was referred on the basis of several image quality criteria. A significant bias in tumour ADC values from DPsti-TSE was present. Further improvements of DPsti-TSE are needed until it can replace DW-EPI.

## Key points

- Diffusion-weighted stimulated-echo turbo spin-echo sequences (DPsti-TSE) provided geometrically less distorted rectal cancer images compared with DW-echo-planar imaging (DW-EPI).
- DPsti-TSE showed a potential for accurate rectal tumour delineation, which is important for rectal cancer radiotherapy planning.
- DPsti-TSE is a promising DWI technique for rectal cancer staging, but further optimisation and validation are still needed. Discrepancies in apparent diffusion coefficient compared to DW-EPI also need to be further investigated.

## Background

Diffusion-weighted imaging (DWI) plays an important role in rectal cancer imaging. As a noninvasive functional magnetic resonance imaging (MRI) technique, DWI is sensitive to extracellular water diffusion motion and discriminates tissues with different cellularity [1]. It thereby provides added value to conventional T2-weighted MRI in rectal cancer detection [2] and improves the diagnostic accuracy in the discrimination between fibrosis and residual tumour after neoadjuvant chemotherapy and radiation therapy [3, 4]. Rectal tumours typically show restricted diffusion, which causes them to show distinctly high signal compared to the suppressed background on DWI at high b-values (> 800 s/mm^2^) [2, 5]. Additionally, the apparent diffusion coefficient (ADC) estimated by DWI provides a quantitative measurement, which could be an objective biomarker for rectal cancer treatment response prediction and evaluation [2, 6].

One of the challenges in rectal cancer DWI, however, is image distortion caused by intra-rectal gas [7]. Most frequently, a two-dimensional (2D) DWI single-shot echo-planar imaging sequence (DW-EPI) is used, because of its short scan time and robustness to motion [8]. However, EPI-based methods are sensitive to susceptibility differences near the gas-filled rectum, which may result in strong geometrical distortions [9]. These image distortions can often lead to signal pileup and inaccurate ADC estimations [7]. Another difficulty for conventional rectal cancer DW-EPI is the low signal-to-noise ratio at high b-values, which may cause ADC underestimation and variation [7, 10].

To reduce the distortions in DW-EPI, the rectum may be filled and/or distended with a susceptibility-matching material [3], or the amount of intra-rectal gas may be reduced with a preparatory micro-enema [11]. However, these preparatory steps are an extra-burden for the patient [6, 12]. The rectum distention could hamper correct assessment of the relation between the rectal tumour and surrounding structures [13]. Fortunately, the geometrical distortions can be mitigated with a non-EPI-based readout. While non-EPI based DWI methods have been applied in other anatomies [14–16], their diagnostic value in rectal cancer DWI has not been investigated yet. In a recent paper [17], we introduced a three-dimensional (3D) diffusion-prepared stimulated-echo turbo-spin-echo sequence (DPsti-TSE), which enables distortion-free DWI, and provided a preliminary demonstration of the possibility of geometrically undistorted DWI of the healthy rectum.

In the present study, we have compared, qualitatively and quantitatively, the image quality of 3D DPsti-TSE and 2D DW-EPI for locally advanced rectal cancer.

## Methods

### Patient population

Thirty-three patients (24 males, 9 females) with locally advanced rectal cancer (≥ cT3cd and/or mesorectal fascia positive [MRF+] and/or cN+) were recruited in this prospective study, prior to treatment. All patients were consecutively recruited within a 5-month time period. No patient exclusion criteria were applied. Their median age was 63 years (range 36–88 years). In locally advanced rectal cancer, the tumour is commonly large and conspicuous, which makes its delineation easy to perform even at low image resolution. To date, both in research and clinics, DWI is most often applied for assessing locally advanced rectal cancer [13]. As such, we started off with this subgroup as a proof of principle. All the patients provided written informed consent. The consent form and study procedures were reviewed and approved by the institutional review board.

### Scan protocol

Patients were scanned in a single institution on one of two 3-T MRI scanners (Ingenia or Achieva, Philips, Best, the Netherlands). Patients received no bowel preparation or spasmolytic drugs before scanning. A posterior and an anterior phase array coil with a total of 24 receive channels was used. The anterior coil was supported by a frame and hovered over the patient’s body to avoid deformation of the abdomen.

All subjects received a 3D DPsti-TSE and a 2D DW-EPI scan of the rectum area. To compare their performance, both sequences were applied with identical voxel size (2.3 × 2.3 × 3.0 mm^3^), scan orientation (axial) and b-values (200 and 1000 s/mm^2^). For the DPsti-TSE sequence, diffusion gradients were applied in the [1, 1, 1] direction in the scanner [x, y, z] coordinate frame. Diffusion gradients were 1st-order gradient moment nulled to reduce sensitivity to the physiological motion [17]. For the DW-EPI sequence, diffusion gradients were applied in three orthogonal directions with no gradient moment nulling. The total scan durations for DPsti-TSE and DW-EPI were 6 min 55 s and 3 min 33 s, respectively. A detailed overview of the scan parameters is reported in Table 1. Pixel-wised ADC maps in the entire field of view were calculated from the DW images, based on the formula $ADC=\ln \left(\frac{S_{b=200}}{S_{b=800}}\right)/\left(800-200\right)$, where *S*_*b*_ is the image intensity. For anatomical reference, a T2-weighted TSE scan without fat suppression was acquired with the same field of view and orientation but with a higher resolution of 0.7 × 0.7 × 3.0 mm^3^.

**Table 1:** Scan parameters

	FOV (AP × RL × FH) (mm3)	Resolution (AP × RL × FH) (mm3)	SENSE	Fat suppression	Scan mode	Echo train length	TR / TE^a^ (ms)	Total scan duration
DW-EPI	256 × 256 × 99	2.3 × 2.3 × 3	2 (RL)	SPAIR	Multislice 2D	55	5469 / 66	3 min 33 s
DPsti-TSE	256 × 264 × 99	2.3 × 2.3 × 3	2 × 1.4(RL × FH)	SPAIR	3D	50	2500 / 18	6 min 55 s
T2W	200 × 281 × 126	0.7 × 0.7 × 3	none	none	Multislice 2D	30	5953 / 120	5 min 33 s
	Profile order	Half scan	Diffusion mixing time (ms)	M1 nulled diffusion gradient	b value (s/mm^2^)	Diffusion direction	Average	
DW-EPI	Linear	0.618	59	No	200, 1000	Orthogonal	4 (b = 200 s/mm2)	
							8 (b = 1000 s/mm2)	
DPsti-TSE	Low-high; Turbo Z	0.8 × 0.9 (RL × FH)	62	Yes	200, 1000	[1, 1, 1]	2 (b = 200 s/mm2)	
							8 (b = 1000 s/mm2)	
T2W	Linear	None	--	--	--	--	1	

*2D* Two-dimensional, *3D* Three-dimensional, *DPsti-TSE* Diffusion-prepared stimulated echo turbo spin-echo, *DW-EPI* Diffusion-weighted echo-planar imaging, *FOV* Field of view, *SENSE* SENSitivity Encoding, *SPAIR* Spectral attenuated inversion recovery fat suppression, *TE* Echo time, *TR* Repeation time

^**a**^TE for DPsti-TSE does not include the diffusion mixing time. TE for DW-EPI measurement includes the diffusion mixing time

To reassure DPsti-TSE can generate correct ADC values as we previously reported [17], we performed a phantom scan with similar scanning parameter as above. The phantom was measured at 0 °C with the 3 T Ingenia scanner.

### Qualitative evaluation

Image quality was independently evaluated by two radiologist experts in pelvic MRI (D. M. J. L. and M. K.) using the RadiAnt DICOM Viewer software (Medixant, Poznan, Poland). Images were presented to the radiologists in two rounds.

In the first round, radiologists were blinded to the specific DWI method, and they received images from different patients and DWI methods in a random order. Images were scored for overall image quality and anatomical detail, with reference to the T2-weighted images for anatomical correlation. A total of nine aspects were scored, six of them regarding image quality (quality of fat suppression, image sharpness, presence of motion artefacts, rectum distortion, signal pileup and overall quality) and three of them regarding anatomical detail (rectal wall visibility on b = 1000 s/mm^2^ images, tumour conspicuity on b = 1000 s/mm^2^ images and tumour conspicuity on ADC maps). Scoring was performed on a 5-point scale ranging from 0, indicating poor quality or conspicuity (not diagnostic images), to 4, indicating excellent quality or conspicuity. More description for the five scales is given in Table 2.

**Table 2:** Description for DWI image quality scales

Scale	Description
0	Image has no diagnosis value^a^; tumour or rectal wall is undetectable.
1	Image quality is unacceptable or insufficient^a^; tumour or rectal wall is poorly visible.
2	Image has moderate quality^a^; tumour or rectal wall is visible but not delineable.
3	Image has good quality^a^; tumour or rectal wall is delineable but without details.
4	Image has excellent quality^a^; tumour or rectal wall is delineable with details.

^a^Six aspects of the image quality are scored. They are fat-suppression, image sharpness, presence of motion artifacts, rectum distortion, signal pile-up and overall quality

In the second scoring round, diffusion images for the same patient acquired by the two different methods were presented to the two radiologists side by side without labelling the method name. Radiologists were asked to indicate strong, moderate or no preference to either of the two images/methods.

### Quantitative evaluation

To evaluate the tumour ADC values obtained with each of the two diffusion sequences, the whole tumour volume was delineated by drawing region of interest (ROIs) on the tumour-containing slices in DPsti-TSE and DW-EPI images separately. To minimise the influence of the T2-weighted images on ROI contours, the ROI drawing was conducted in two steps. First, a circle was drawn around the tumour in every slice using the T2-weighted images as reference, to only roughly indicate the tumour location. In the second step, the tumour ROI was delineated within the circle using the DW images only. The ROIs were then copied to corresponding ADC maps to extract tumour ADC values.

**Fig. 1:**
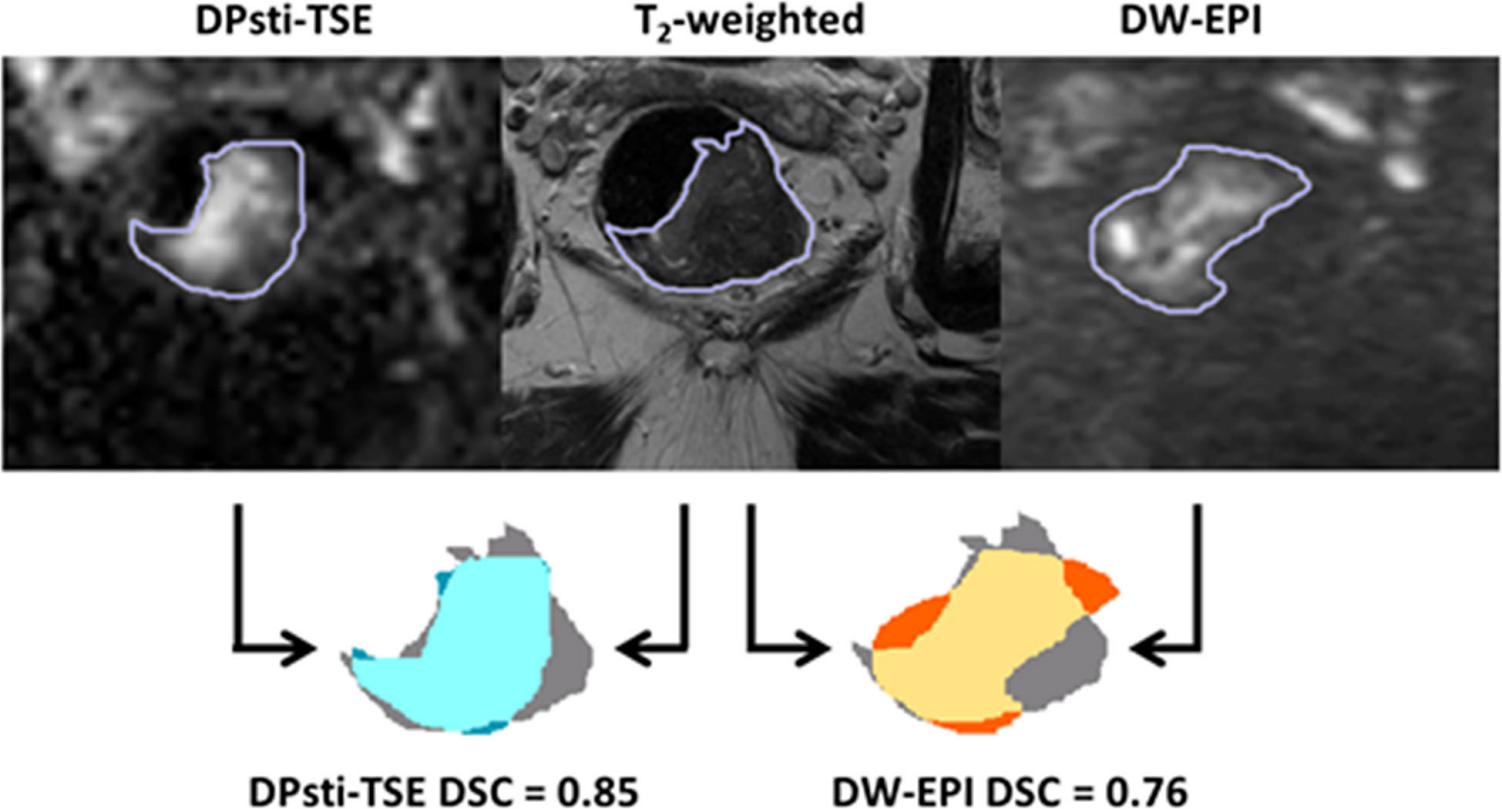
Illustration of the DSC calculation. First, tumour ROIs for a rectal motion-free slice (b = 1000 s/mm^2^) were delineated using the two-step drawing approach. DSC values were then calculated for DPsti-TSE ROI and DW-EPI ROI separately. The DSC value is the ratio of the overlapped area to the mean area of two ROIs, from a DW image and the T2-weighted image, respectively. (*DPsti-TSE* diffusion-prepared stimulated echo turbo spin-echo, *DSC* Dice similarity coefficient, *DW-EPI* diffusion-weighted echo-planar imaging, *ROI* region of interest)

To evaluate the image distortions, one rectal motion-free slice location for each patient was chosen. Tumour ROIs on T2-weighted images for the same locations were delineated as a reference and compared with the ROIs drawn on DPsti-TSE and DW-EPI images. Specifically, the tumour ROI Dice similarity coefficient (DSC) for DPsti-TSE and DW-EPI was compared. DSC was used to quantify the similarity between ROI from DW images and the ROI from T2-weighted images [18]. DSC values were defined as the ratio between the overlapped area and the average area of two ROIs, where a DSC value of 1 indicates perfect alignment of ROIs and a value of 0 means no overlap of the two ROIs. The DSC comparison is illustrated in Fig. 1. To assess the ROI drawing reliability, all ROI delineations for tumour distortion evaluation were repeated after 1 month.

Tumour delineation was performed by a research assistant under the supervision of M.K. The quantitative evaluation was performed by an in-house-built software programmed in MATLAB (the MathWorks, Natick, MA).

### Statistical analysis

All statistical analyses were performed using the Statistical Package for the Social Sciences (SPSS, version 24, Inc., Chicago, IL, USA). The level of significance was set to 0.05. For image quality evaluation, the consistency between both radiologists was determined by calculating the intraclass correlation coefficient (ICC). According to Landis and Koch’s guideline [19], ICC lower than 0.20 was considered poor, from 0.21 to 0.40 fair, from 0.41 to 0.60 moderate, from 0.61 to 0.80 good and higher than 0.80 excellent. Scores from both radiologists were then averaged, and a Wilcoxon signed-rank test was performed to test whether there were significant differences between DPsti-TSE and DW-EPI for each evaluated parameter.

A paired t-test was performed to compare tumour ADC values between the DPsti-TSE and DW-EPI methods. For the quantitative tumour distortions evaluation, the agreement of DSC values from two ROI delineation repetitions was determined by the ICC value. DSC values from two repetitions were averaged. A paired t-test was performed to determine if DCS values from both methods were significantly different. For both ADC and DCS values, the Kolmogorov–Smirnov test (significant level at 0.05) was performed for data normality check.

## Results

### Image quality evaluation

**Fig. 2:**
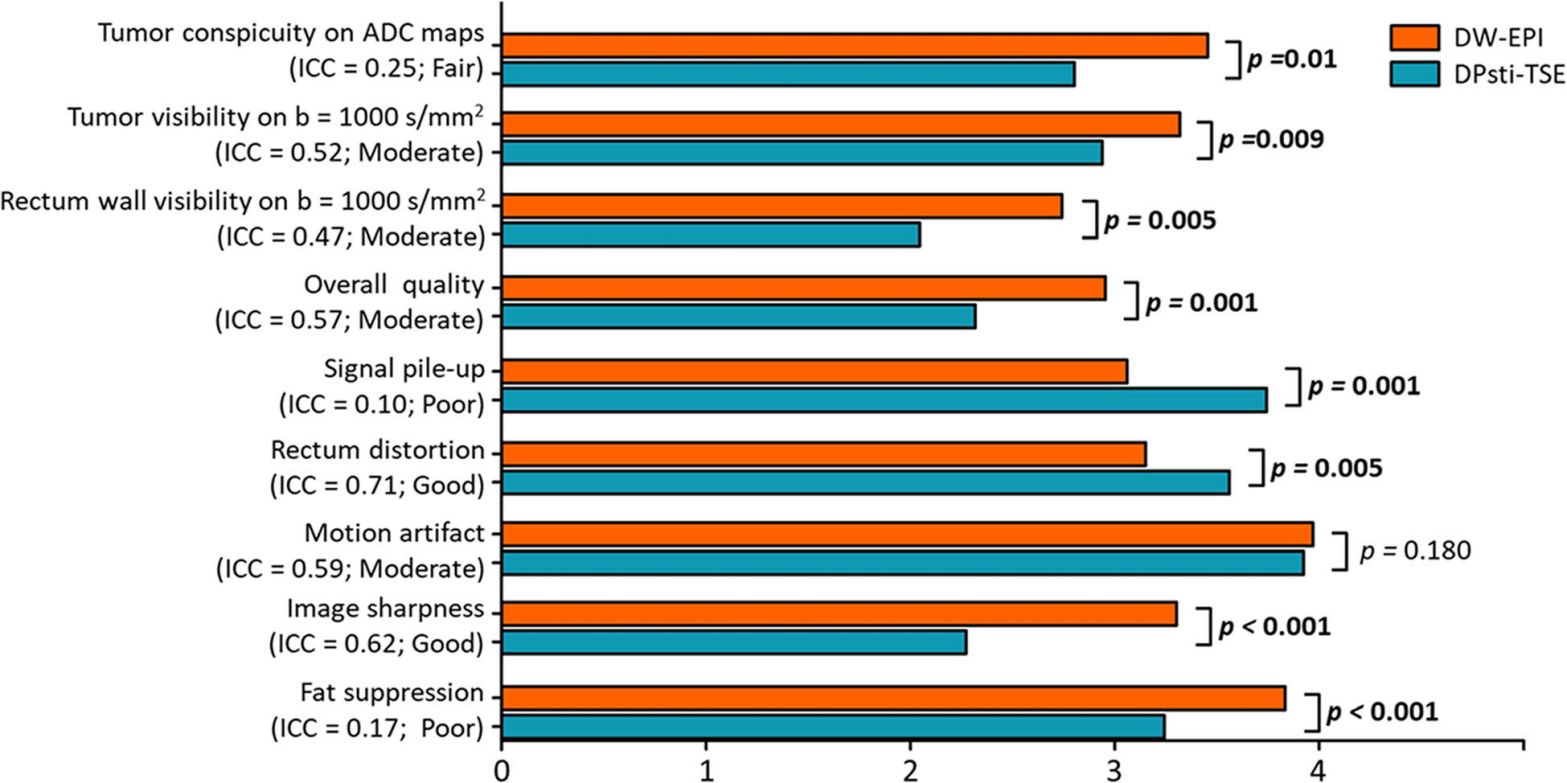
The mean scores for DW-EPI and DPsti-TSE images, averaged over all patients and observers. For each parameter, the ICC value between two observers is shown in parentheses. (*DPsti-TSE* diffusion-prepared stimulated echo turbo spin-echo, *DW-EPI* diffusion-weighted echo-planar imaging, *ICC* intraclass correlation coefficient)

Radiologists’ scoring results are summarised in Fig. 2. The bar plot shows the scores for the DPsti-TSE and DW-EPI methods for the six image quality aspects and the three anatomical detail aspects, as well as the ICC values for two radiologists. Overall, DW-EPI obtained significantly higher scores for tumour conspicuity on ADC maps (*p =* 0.01), tumour visibility on b = 1000 s/mm^2^ (*p =* 0.009) and rectum wall visibility on b = 1000 s/mm^2^ (*p =* 0.005). DW-EPI also scored significantly higher on overall image quality (*p =* 0.001), image sharpness (*p <* 0.001) and quality of fat suppression (*p <* 0.001). The DPsti-TSE method had significantly better performance in terms of signal pileup (*p =* 0.001) and rectum distortion (*p =* 0.005). The two methods did not differ significantly with respect to presence of motion artefacts (*p =* 0.180). Score distributions for each parameter can be found in the supplementary material (Additional file 1: Figure S1).

When comparing the DPsti-TSE and DW-EPI images side-by-side, DPsti-TSE was preferred by the radiologists in 12 cases (3 cases with strong preference), whereas DW-EPI was preferred in 17 cases (3 cases with strong preference). In the remaining four cases, the radiologists expressed no preference for either technique. The inter-reader agreement for the preference scoring was moderate (ICC = 0.51).

**Fig. 3:**
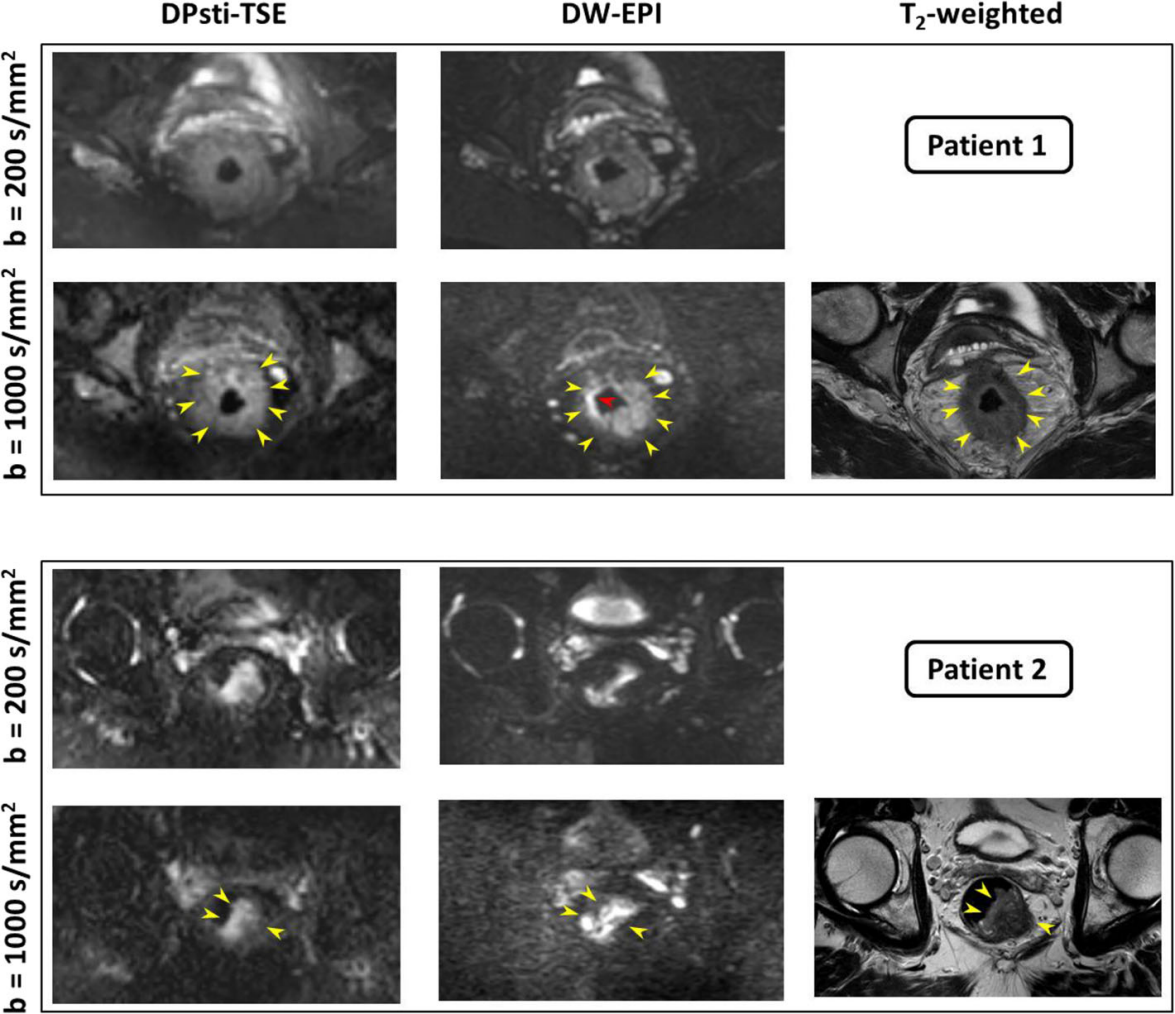
Representative images for two locally advanced rectal cancer patients. Tumours are indicated by yellow arrows, which are hyperintense on the b = 1000 s/mm^2^ images and hypointense on T2-weighted images. Intra-rectal gas led to minor signal pileup in DW-EPI images for patient 1 (red arrow) but more severe tumour distortion in DW-EPI images for patient 2. (*DPsti-TSE* diffusion-prepared stimulated echo turbo spin-echo, *DW-EPI* diffusion-weighted echo-planar imaging)

Representative DW images from DPsti-TSE and DW-EPI with a T2-weighted anatomical reference are shown in Fig. 3 for two patients. Intra-rectal gas was present in both cases, which led to minor signal pileup in DW-EPI images for patient 1 (red arrow) and severe tumour distortion in DW-EPI images for patient 2. In contrast, in the DPsti-TSE images, no signal pileup or tumour distortion was observed. Overall, DW-EPI images appeared sharper than DPsti-STE.

### Quantitative evaluation

**Fig. 4:**
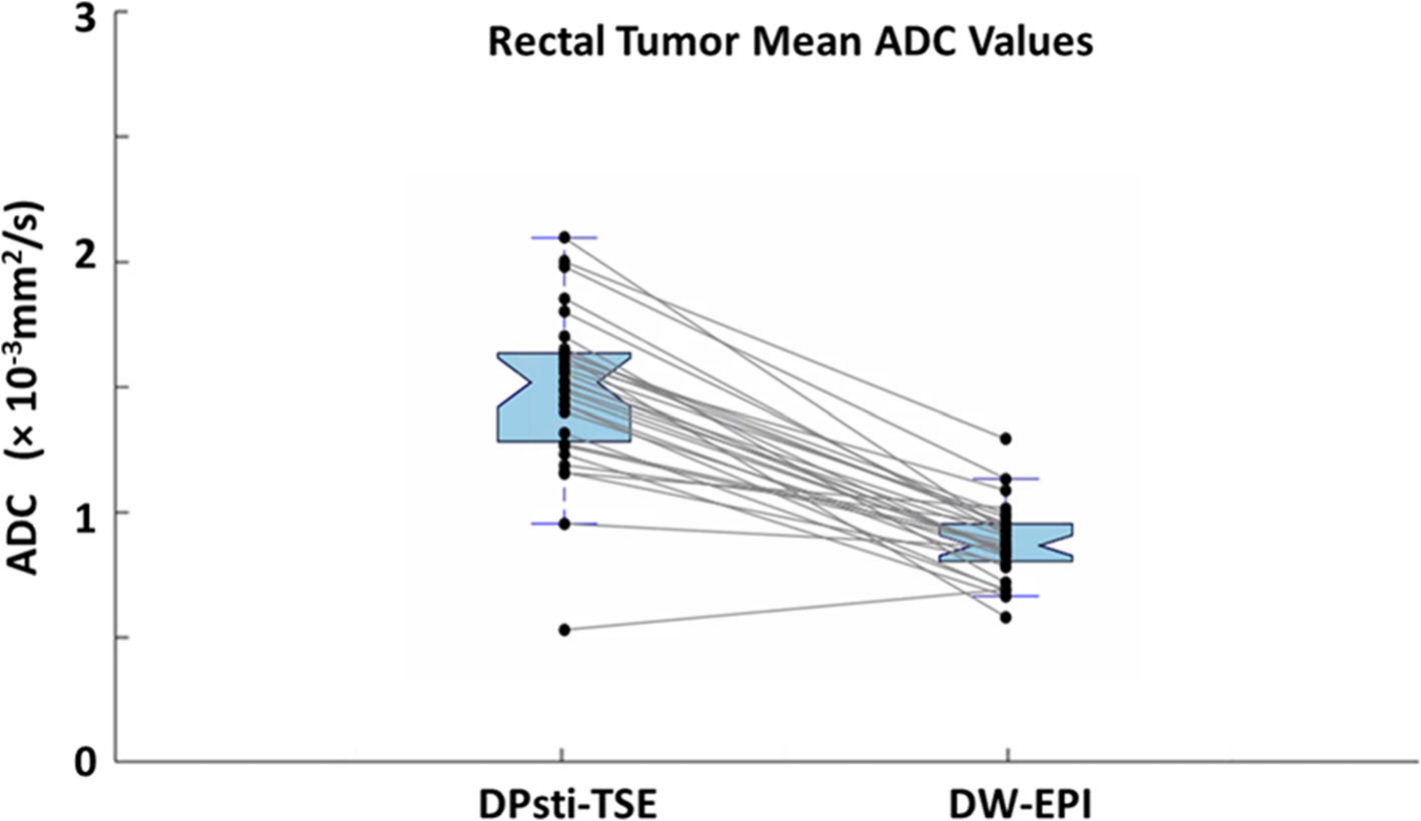
Tumour ADC values for 31 patients obtained by DPsti-TSE and DW-EPI methods. The box plots show the 75th, 50th and 25th percentile of ADC values for all 29 patients. For the same patients, ADC values from DPsti-TSE and DW-EPI are connected. The DPsti-TSE method generated higher tumour ADC values than the DW-EPI method: (1.47 ± 0.32) × 10^−3^ mm^2^/s and (0.86 ± 0.15) × 10^−3^ mm^2^/s, respectively (*p <* 0.001). (*ADC* apparent diffusion coefficient, *DPsti-TSE* diffusion-prepared stimulated echo turbo spin-echo, *DW-EPI* diffusion-weighted echo-planar imaging)

**Fig. 5:**
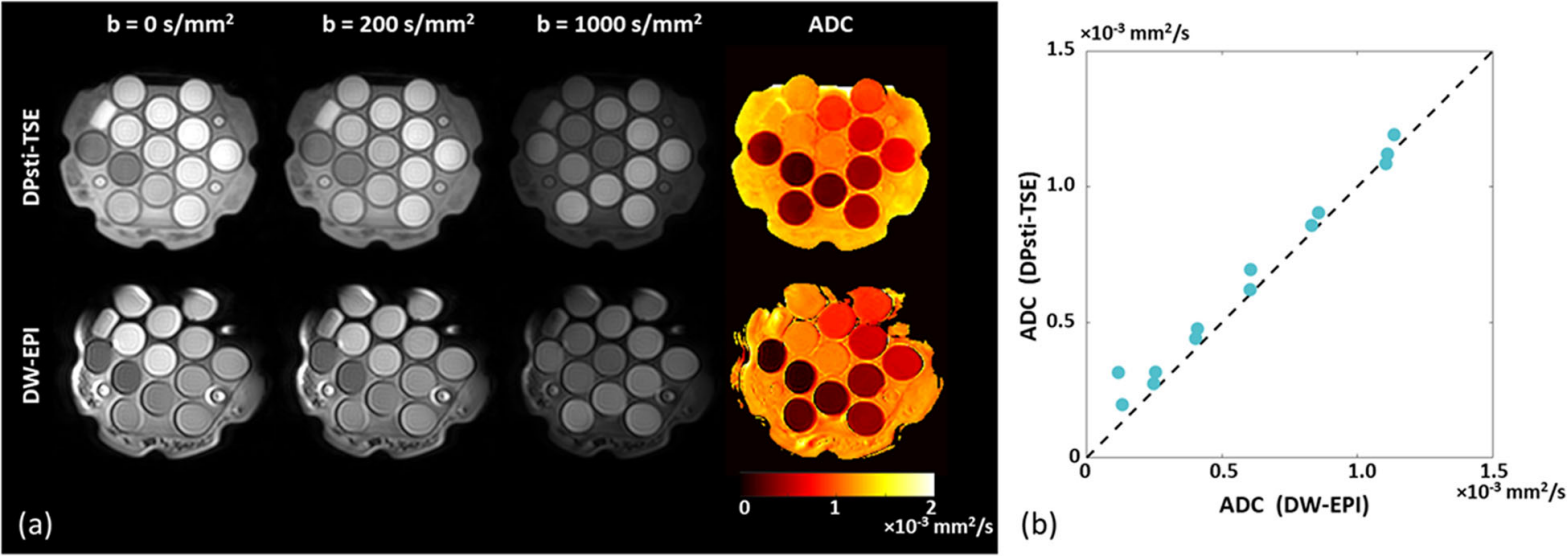
Diffusion phantom measurement. **a** DWI images and ADC maps for the diffusion phantom obtained using DPsti-TSE and DW-EPI methods. **b** The comparison of ADC mean values obtained from DPsti-TSE and DW-EPI for 13 tubes contained in the phantom. Identity line (45° line) was plotted for reference. (*ADC* apparent diffusion coefficient, *DPsti-TSE* diffusion-prepared stimulated echo turbo spin-echo, *DW-EPI* diffusion-weighted echo-planar imaging)

Of the 33 cases, 31 were included for the rectal tumour volume ROI drawing. Two cases were excluded due to unacceptable image quality for both diffusion methods. In Fig. 4, the comparison for the rectal tumour mean ADC values was obtained by the DPsti-TSE and DW-EPI methods. The mean ADC value from DPsti-TSE was markedly and significantly higher than the values from DW-EPI (1.47 ± 0.32 *versus* 0.86 ± 0.15 × 10^−3^ mm^2^/s, *p <* 0.001). The Kolmogorov–Smirnov test did not reject the data normality assumption for tumour mean ADC values. The good agreement of the ADC values from DPsti-TSE compared with DW-EPI in the phantom scan is shown in Fig. 5.

**Fig. 6:**
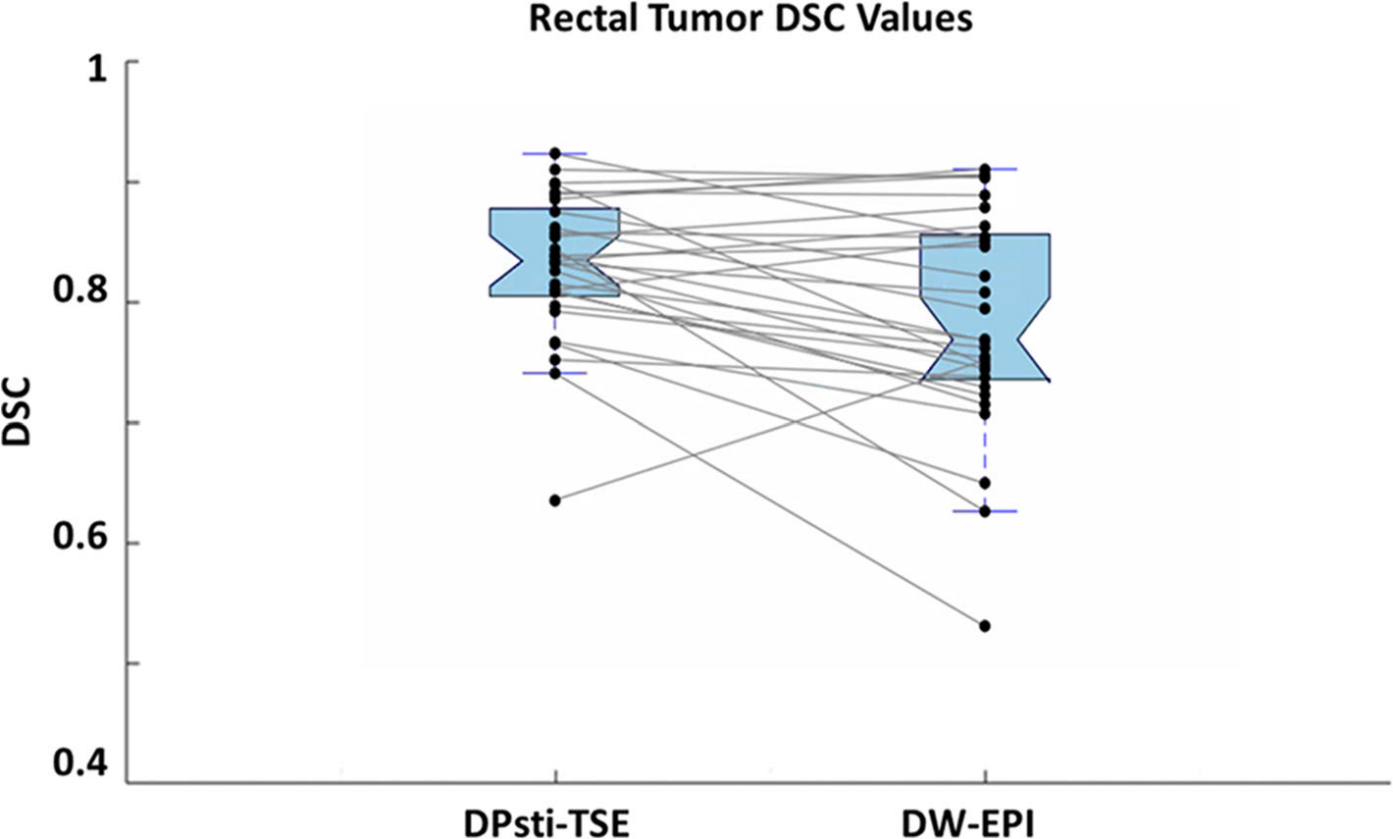
Comparison of tumour region-of-interest DSC values for DPsti-TSE and DW-EPI methods. The box plots show the 75th, 50th, and 25th percentile of DSC values for all 29 patients. For the same patients, DSC values from DPsti-TSE and DW-EPI are connected. DSC values for DPsti-TSE were significantly higher than DSC values from DW-EPI (*p =* 0.010). (*DPsti-TSE* diffusion-prepared stimulated echo turbo spin-echo, *DSC* Dice similarity correlation coefficient, *DW-EPI* diffusion-weighted echo-planar imaging)

For the tumour DSC comparison, 29 cases were included, for which slice locations with no rectal motion could be found. The comparison of rectal tumour DSC values from DPsti-TSE and DW-EPI methods is shown in Fig. 6. In 21 of the 29 cases, the DSC value for DPsti-TSE was higher than that of DW-EPI. A paired t-test showed that DSC values from DPsti-TSE (0.84 ± 0.06) were significantly higher (*p =* 0.010) than those from DW-EPI (0.80 ± 0.10). The repeated tumour ROI delineation generated similar DSC values with excellent agreement (ICC = 0.82). The Kolmogorov–Smirnov test did not reject the data normality assumption for DSC values. Tumour ROIs for all cases are shown in the supplementary material (Additional file 1: Figure S2).

## Discussion

This study investigated the feasibility of the DPsti-TSE method in rectal tumour DWI. To the best of our knowledge, this is the first study to perform a within-patient comparison of a TSE-based DWI method to a standard DW-EPI method, both qualitatively and quantitatively. DPsti-TSE demonstrated its ability for 3D distortion-free rectal tumour DWI. Compared to DW-EPI, DPsti-TSE results in a significant decrease in rectum distortion and signal pileup. However, the method scored lower in other image quality and anatomical detail aspects as compared to standard DW-EPI. Moreover, DPsti-TSE resulted in much higher tumour ADC values as compared to DW-EPI.

Accurate geometrical information on the rectum and tumour is of great importance for radiotherapy planning [20, 21]. Previously, Burbach et al. [20] demonstrated that rectal cancer diffusion-weighted images from TSE-based sequences registered well with T2-weighted images, leading to improved inter-observer agreement of tumour delineation. In contrast, registration of DW-EPI to T2-weighted images was more challenging [20, 22]. Van Griethuysen et al. [11] recently demonstrated that by using a preparatory micro-enema shortly before rectal DW-EPI, both the incidence and severity of gas-induced artefacts can be significantly reduced. However, to the best of our knowledge, this procedure is not yet widely adopted as a clinical routine [13]. In this study, without bowel preparation, we found that DPsti-TSE suffered less from tumour distortion in comparison with DW-EPI. It is worth noting that the DW-EPI scanning protocol used in this study was optimised to reduce susceptibility distortions by selecting a short EPI echo train length enabled by means of parallel imaging, half scan and REST slab fold-over suppression.

DPsti-TSE scored lower for image sharpness. This was primarily caused by the long TSE echo train (50 echoes), which generated strong T2-decay modulation across k-space leading to image blurring [23, 24]. We used a long TSE echo train to enhance the TSE readout efficiency for the 3D acquisition and avoid long scan times. This blurring is likely the cause why the DPsti-TSE scored lower on rectum wall and tumour visibility. In future implementations, the resolution of DPsti-TSE can be improved with shorter TSE echo train length, in combination with advanced acceleration techniques such as compressed sensing and reduced field of view to prevent long scan durations.

DPsti-TSE is capable to achieve a much higher resolution than DW-EPI with more shots at the expense of longer scan time, as we demonstrated in an earlier study [17]. Although the in-plane resolution of 2.3 × 2.3 mm^2^ was adequate in this study, higher resolution DPsti-TSE images may be necessary in certain applications, such as after chemoradiotherapy when the rectal tumour size may be small and visualisation on DW-EPI becomes very challenging [3, 25].

DPsti-TSE also scored lower on fat suppression performance compared to DW-EPI. We believe this was due to variations in the B_0_ shimming performance, which is crucial for the spectral attenuated inversion recovery fat suppression technique [26, 27]. However, unlike in DW-EPI images, the remaining fat signal in DPsti-TSE images should not lead to problematic chemical shift artefacts, as the TSE readout has a high bandwidth for the phase encoding direction [28].

Rectal tumour ADC is considered a promising imaging biomarker for tumour diagnosis and treatment evaluation [3, 29]. We found tumour ADC values from DW-EPI in accordance with ADC values for rectal tumours as reported in previous studies [7, 12, 25, 30]. However, tumour ADC values from the DPsti-TSE method were much higher compared to the DW-EPI. This was unexpected given the good ADC agreement in the phantom scan. A possible explanation for the discrepancy in ADC values may be found in physiological motion during the in vivo scan. Motion leads to blurring and signal voids by phase incoherence between shots, which may cause ADC overestimation [1, 31]. We recently introduced a 3D navigator technique to correct for phase incoherence between shots in 3D DPsti-TSE [32]; however this was not yet applied in the current study. Another important factor may be the difference in diffusion gradients between DPsti-TSE and DW-EPI. DPsti-TSE contains M1 nulled diffusion gradients in one direction, while DW-EPI applies non-M1 nulled gradients in three orthogonal directions. Due to the M1 nulling, signal from blood in the tumour may have been partially preserved at lower b-value leading to a stronger decay of the signal at high b-value and consequently a higher estimated ADC value. In DW-EPI, the blood signal does not contribute for either b = 200 s/mm^2^ nor 1000 s/mm^2^ [33]. Finally, underestimation of ADC values by DW-EPI due to low signal-to-noise ratio could be another factor. The existence of a significant noise contribution can be verified from the surrounding muscle area in the b = 1000 s/mm2 DW-EPI images (Fig. 3). Noise leads to overestimation of signal at b = 1000 s/mm^2^, leading to lower ADC values [10]. The true reason for the higher ADC values measured with DPsti-STE and how this affects the use of ADC as tumour biomarker remain to be investigated. Meanwhile, more extensive research will be required to establish standardised ADC values for DPsti-TSE, their reproducibility and appropriate cut-offs, before they can be used as a potential biomarker.

This study has some limitations. First, tumour ADC values were not correlated to clinical diagnosis or histological tumour grading. Second, imaging was done without any bowel preparation, such as rectal filling or a preparatory micro-enema, to reduce the amount of gas in the rectal lumen. The images were therefore relatively prone to gas-induced susceptibility artefacts. Although this may be considered a limitation, it on the other hand provides a good opportunity to study the potential beneficial effects of DPsti-TSE to reduce these types of artefacts. Moreover, it represents common clinical practice where rectal DWI is still performed without bowel preparation in the majority of centres [13]. Third, delineation of tumour volume ROIs for the ADC quantification was only done once. Therefore, repeatability of tumour volume delineation was not assessed. In this respect, automatic rectum tumour segmentation with satisfactory robustness and accuracy can provide objective analyses for both tumour ADC and DSC value comparisons. Fourth, the inter-reader ICC values for some qualitative evaluation scoring aspects were low, which weakened corresponding comparison statements such as for signal pileup and fat suppression performances in diffusion-weighted images. Fifth, although we perform blinded comparisons in this study, radiologists reported that they could still distinguish the two methods due to obvious differences in the image appearance. They might unintentionally preferred DW-EPI images as they were accustomed to this type of images from the clinical routine. Finally, our cohort included only locally advanced rectal cancer cases, which are typically relatively large and may therefore be more conspicuous. Although this represents the subgroup of rectal cancer in which DWI is to date most commonly used, further research will also need to include smaller tumours and restaging settings to assess the general applicability DPsti-TSE in rectal cancer.

In conclusion, the DPsti-TSE method provided geometrically less distorted rectal cancer diffusion images. In comparison with a standard DW-EPI method, DPsti-TSE demonstrated significantly less image distortion and signal pileup. DPsti-TSE therefore may provide added value for improving rectal tumour delineation. However, it is currently limited by longer acquisition times, and its diagnostic image quality was not favoured over DWI-EPI by the radiologists in our study. Furthermore, a significant bias in quantitative parameters may be present. Further research and improvements to DPsti-TSE are needed until it can replace DW-EPI.

## Supplementary information

**Additional file 1: Figure S1.** Score distributions for all evaluation aspects. **Figure S2.** Tumor ROIs from the first delineation of 29 cases used for the DSC calculation. DSC calculation is illustrated in Fig. 1. DSC values derived from two ROI delineations had excellent agreement (ICC = 0.82).

## Data Availability

The datasets used and analysed during the current study are available from the corresponding author on reasonable request.

## Abbreviations

2D: Two dimensional
3D: Three dimensional
ADC: Apparent diffusion coefficient
DSC: Dice similarity coefficient
DW-EPI: Diffusion-weighted echo-planar imaging
DWI: Diffusion-weighted imaging
EPI: Echo-planar imaging
ICC: Intraclass correlation coefficient
MRI: Magnetic resonance imaging
ROI: Region of interest

## References

[1] Bammer R (2003). Basic principles of diffusion-weighted imaging. Eur J Radiol.

[2] Rao SX, Zeng MS, Chen CZ (2008). The value of diffusion-weighted imaging in combination with T2-weighted imaging for rectal cancer detection. Eur J Radiol.

[3] Kim SH, Lee JM, Hong SH (2009). Locally advanced rectal cancer: added value of diffusion-weighted MR imaging in the evaluation of tumor response to neoadjuvant chemo-and radiation therapy. Radiology.

[4] Lambregts DM, Vandecaveye V, Barbaro B (2011). Diffusion-weighted MRI for selection of complete responders after chemoradiation for locally advanced rectal cancer: a multicenter study. Ann Surg Oncol.

[5] Dzik-Jurasz A, Domenig C, George M (2002). Diffusion MRI for prediction of response of rectal cancer to chemoradiation. Lancet.

[6] Intven M, Reerink O, Philippens ME (2013). Diffusion-weighted MRI in locally advanced rectal cancer. Strahlenther Onkol.

[7] Intven M, Reerink O, Philippens ME (2014). Repeatability of diffusion-weighted imaging in rectal cancer. J Magn Reson Imaging.

[8] Charles-Edwards EM, deSouza NM (2006). Diffusion-weighted magnetic resonance imaging and its application to cancer. Cancer Imaging.

[9] Le Bihan D, Poupon C, Amadon A, Lethimonnier F (2006). Artifacts and pitfalls in diffusion MRI. J Magn Reson Imaging.

[10] Dietrich O, Heiland S, Sartor K (2001). Noise correction for the exact determination of apparent diffusion coefficients at low SNR. Magn Reson Med.

[11] van Griethuysen JJ, Bus EM, Hauptmann M (2018). Gas-induced susceptibility artefacts on diffusion-weighted MRI of the rectum at 1.5 T–Effect of applying a micro-enema to improve image quality. Eur J Radiol.

[12] Lambregts DM, Beets GL, Maas M (2011). Tumour ADC measurements in rectal cancer: effect of ROI methods on ADC values and interobserver variability. Eur Radiol.

[13] Beets-Tan RG, Lambregts DM, Maas M (2018). Magnetic resonance imaging for clinical management of rectal cancer: updated recommendations from the 2016 European Society of Gastrointestinal and Abdominal Radiology (ESGAR) consensus meeting. Eur Radiol.

[14] Deng J, Virmani S, Young J (2008). Diffusion-weighted PROPELLER MRI for quantitative assessment of liver tumor necrotic fraction and viable tumor volume in VX2 rabbits. J Magn Reson Imaging.

[15] Bieri O, Ganter C, Scheffler K (2012). Quantitative in vivo diffusion imaging of cartilage using double echo steady-state free precession. Magn Reson Med.

[16] Gibbons EK, Vasanawala SS, Pauly JM, Kerr AB (2018). Body diffusion-weighted imaging using magnetization prepared single-shot fast spin echo and extended parallel imaging signal averaging. Magn Reson Med.

[17] Zhang Q, Coolen BF, Versluis MJ, Strijkers GJ, Nederveen AJ (2017). Diffusion-prepared stimulated-echo turbo spin echo (DPsti-TSE): an eddy current-insensitive sequence for three-dimensional high-resolution and undistorted diffusion-weighted imaging. NMR Biomed.

[18] Dice LR (1945). Measures of the amount of ecologic association between species. Ecology.

[19] Landis J. Richard, Koch Gary G. (1977). The Measurement of Observer Agreement for Categorical Data. Biometrics.

[20] Burbach JP, Kleijnen JP, Reerink O (2016). Inter-observer agreement of MRI-based tumor delineation for preoperative radiotherapy boost in locally advanced rectal cancer. Radiother Oncol.

[21] Ling CC, Humm J, Larson S (2000). Towards multidimensional radiotherapy (MD-CRT): biological imaging and biological conformality. Int J Radiat Oncol Biol Phys.

[22] Regini F, Gourtsoyianni S, De Melo RC (2014). Rectal tumour volume (GTV) delineation using T2-weighted and diffusion-weighted MRI: implications for radiotherapy planning. Eur J Radiol.

[23] Busse RF, Hariharan H, Vu A, Brittain JH (2006). Fast spin echo sequences with very long echo trains: design of variable refocusing flip angle schedules and generation of clinical T2 contrast. Magn Reson Med.

[24] Mugler JP (2014). Optimized three-dimensional fast-spin-echo MRI. J Magn Reson Imaging.

[25] Barbaro B, Vitale R, Valentini V (2012). Diffusion-weighted magnetic resonance imaging in monitoring rectal cancer response to neoadjuvant chemoradiotherapy. Intern Int J Radiat Oncol Biol Phys.

[26] Dietrich O, Reiser MF, Schoenberg SO (2008). Artifacts in 3-T MRI: physical background and reduction strategies. Eur J Radiol.

[27] Brandão S, Nogueira L, Matos E (2015). Fat suppression techniques (STIR vs. SPAIR) on diffusion-weighted imaging of breast lesions at 3.0 T: preliminary experience. Radiol Med.

[28] BERNSTEIN MATT A., KING KEVIN F., ZHOU XIAOHONG JOE (2004). TOOLS. Handbook of MRI Pulse Sequences.

[29] Curvo-Semedo L, Lambregts DM, Maas M, Beets GL, Caseiro-Alves F, Beets-Tan RG (2012). Diffusion-weighted MRI in rectal cancer: apparent diffusion coefficient as a potential noninvasive marker of tumor aggressiveness. J Magn Reson Imaging.

[30] Lambrecht M, Vandecaveye V, De Keyzer F (2012). Value of diffusion-weighted magnetic resonance imaging for prediction and early assessment of response to neoadjuvant radiochemotherapy in rectal cancer: preliminary results. Int J Radiat Oncol Biol Phys.

[31] Wu W, Miller KL (2017). Image formation in diffusion MRI: a review of recent technical developments. J Magn Reson Imaging.

[32] Zhang Q, Coolen BF, Nederveen AJ, Strijkers GJ (2018). Three-dimensional diffusion imaging with spiral encoded navigators from stimulated echoes (3D-DISPENSE). Magn Reson Med.

[33] Aliotta E, Wu HH, Ennis DB (2017). Convex optimized diffusion encoding (CODE) gradient waveforms for minimum echo time and bulk motion–compensated diffusion-weighted MRI. Magn Reson Med.

