# Supplementary material for: Locally advanced rectal cancer: 3D diffusion-prepared stimulated-echo turbo spin-echo versus 2D diffusion-weighted echo-planar imaging
Source: Eur Radiol Exp. 2020 Feb 7;4:9. doi: 10.1186/s41747-019-0138-x (PMC7005244; doi:10.1186/s41747-019-0138-x)
Supplement: Supplementary file 1 — Additional file 1: Figure S1. Score distributions for all evaluation aspects. Figure S2. Tumor ROIs from the first delineation of 29 cases used for the DSC calculation. DSC calculation is illustrated in Fig. 1. DSC values derived from two ROI delineations had excellent agreement (ICC = 0.82). [file 41747_2019_138_MOESM1_ESM.doc]

# Supplementary material for “Locally advanced rectal cancer: 3D diffusion-prepared stimulated-echo turbo spin-echo versus 2D diffusion-weighted echo-planar imaging”

|  |  |
| --- | --- |


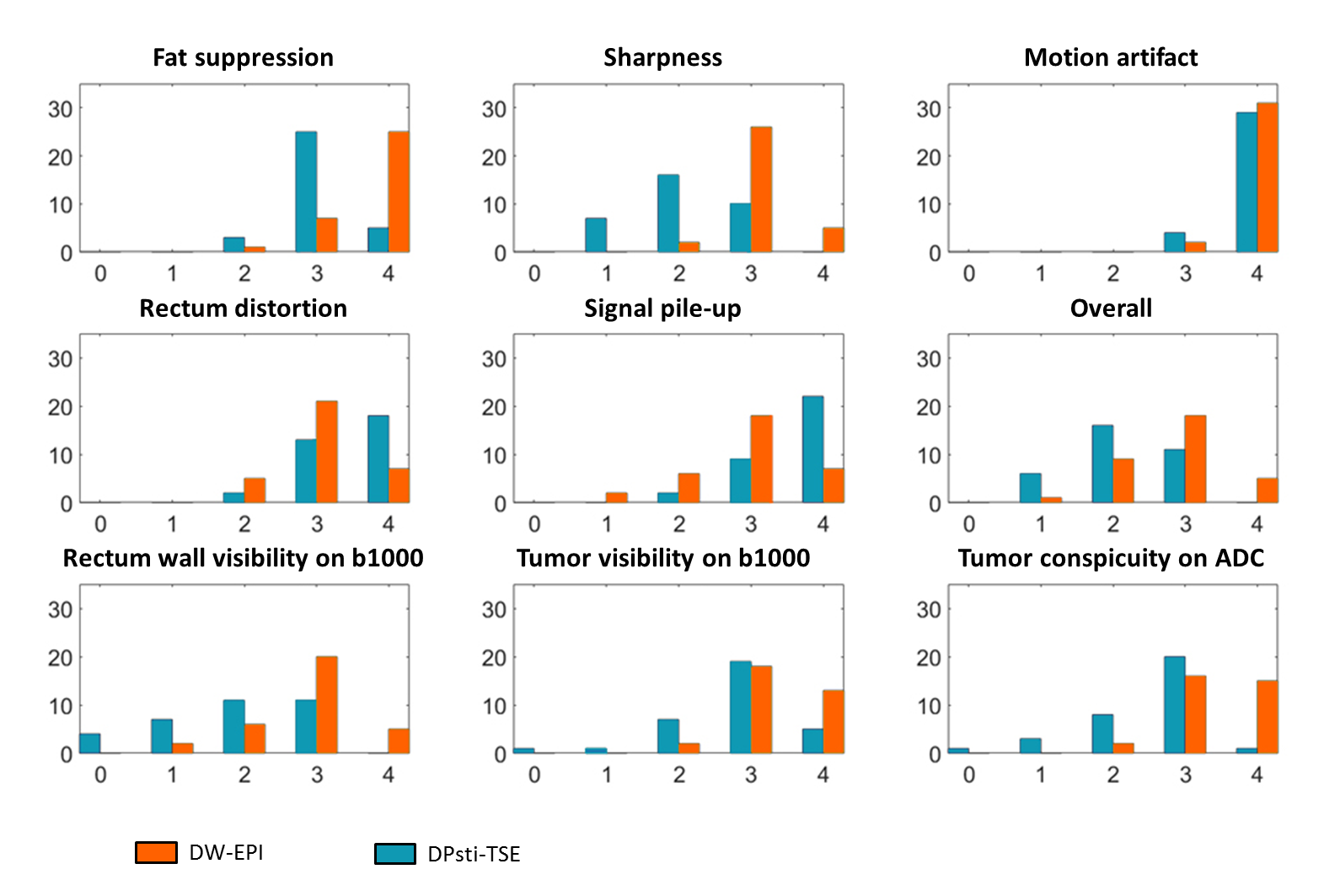


**Figure S1**: Score distributions for all evaluation aspects.


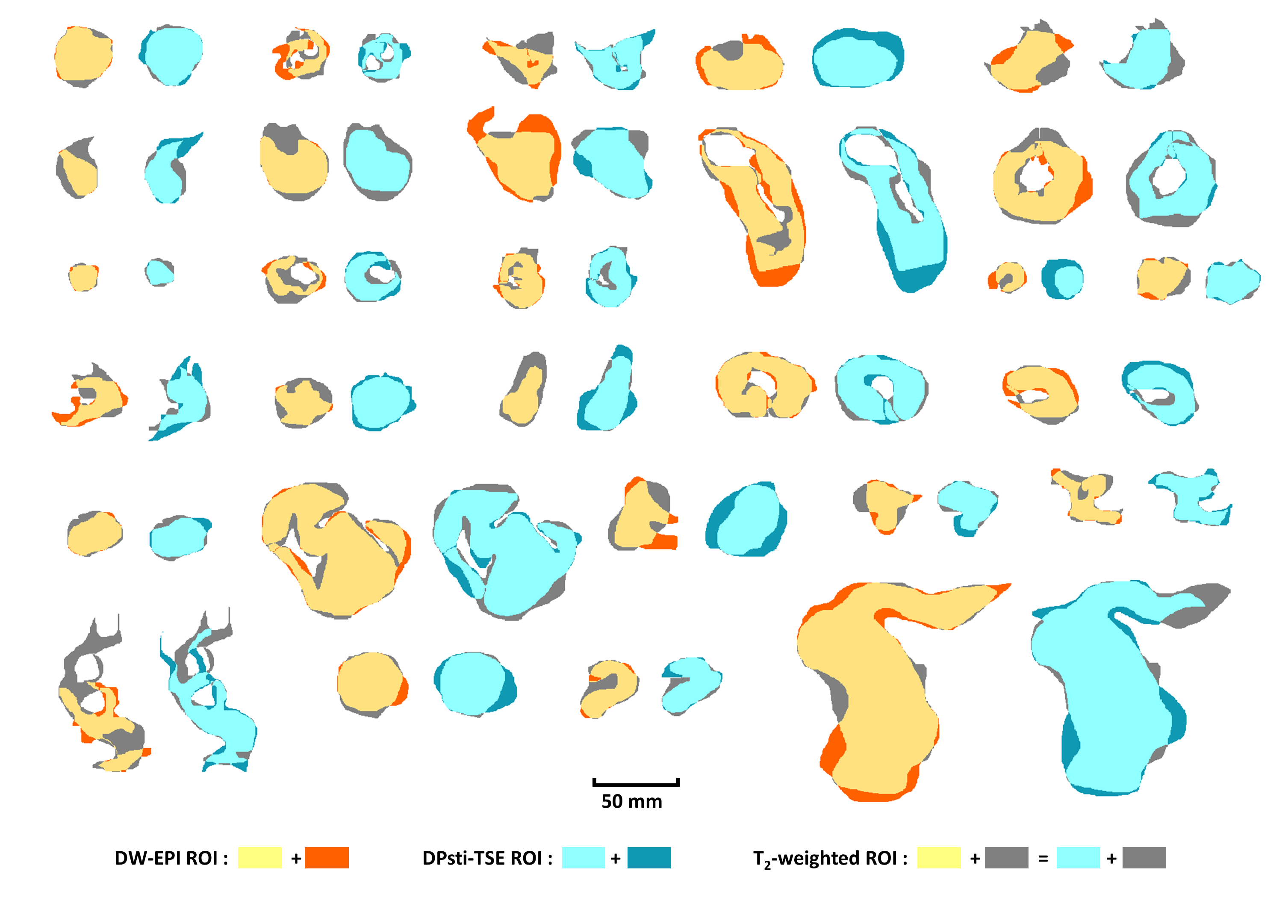


**Figure S2**: Tumor ROIs from the first delineation of 29 cases used for the DSC calculation. DSC calculation is illustrated in **Figure 1**. DSC values derived from two ROI delineations had excellent agreement (ICC = 0.82).
